# NF-κB-Targeted Anti-Inflammatory Activity of *Prunella vulgaris* var. *lilacina* in Macrophages RAW 264.7

**DOI:** 10.3390/ijms141121489

**Published:** 2013-10-30

**Authors:** Yu-Jin Hwang, Eun-Ju Lee, Haeng-Ran Kim, Kyung-A Hwang

**Affiliations:** 1 Department of Agrofood Resources, National Academy of Agricultural Science, RDA, Suwon, Gyeonggi-do 441-853, Korea; E-Mails: yujinh21@skku.edu (Y.-J.H.); bono2180@naver.com (E.-J.L.); kimhrr@korea.kr (H.-R.K.); 2 Department of Biotechnology & Bioengineering, Sungkyunkwan University, Suwon, Gyeonggi-do 440-746, Korea

**Keywords:** *Prunella vulgaris* var. *lilacina*, anti-inflammation, NO, PGE2, NF-κB

## Abstract

*Prunella vulgaris* var. *lilacina*, a herbal medicine, has long been used in Korea for the treatment of sore throat, and to alleviate fever and accelerate wound healing. Although the therapeutic effect of *P. vulgaris* var. *lilacina* is likely associated with anti-inflammatory activity, the precise underlying mechanisms are largely unknown. Here, we sought to elucidate the possible mechanisms of the anti-inflammatory activity. We have investigated the anti-inflammatory activity of the various solvent fractions (hexane, butanol, chloroform and water) from the ethanol extract of *P. vulgaris* var. *lilacina* in activated macrophages. The hexane fraction exhibited higher anti-inflammatory activities, inducing inhibition of nitric oxide and prostaglandin E2 production as well as inducible nitric oxide synthase, cyclooxygenase-2, and tumor necrosis factor-α mRNA expression in response to lipopolysaccharide stimulation. Moreover, the hexane fraction from *P. vulgaris* var. *lilacina* significantly inhibited the activation of the nuclear factor kappa-light-chain-enhancer of activated B cells (NF-κB) and the nuclear translocation of the NF-κB p50 and p65 subunits. These results indicate that *P. vulgaris* var. *lilacina* has an anti-inflammatory capacity *in vitro*, suggesting that it could be a potential source of natural anti-inflammatory agents.

## 1. Introduction

Inflammation is the primary response to infection or injury and is critical for both innate and adaptive immunity [1,2]. Inflammation can be measured by the use of various markers such as nuclear factor kappa-light-chain-enhancer of activated B cells (NF-κB), tumor necrosis factor-α (TNFα), cyclooxygenase (COX), and nitric oxide (NO). In particular, NF-κB is an important transcription factor that induces the transcription of proinflammatory mediators, such as inducible nitric oxide synthase (iNOS) and COX-2, which are involved in the activation of inflammatory and immune responses [3–5]. Since activation of NF-κB leads to inflammation that in turn is involved in the pathogenesis of many diseases such as asthma, rheumatoid arthritis, and inflammatory bowel disease [6], much attention has focused on the development of anti-inflammatory drugs targeting NF-κB [7].

Due to the resistance of such diseases to conventional treatment, as well as the side effects of presently available anti-inflammatory drugs, there is a pressing need for the development of novel anti-inflammatory drugs. In this regard, recent efforts are focusing on finding natural products that show anti-inflammatory properties [8]. Several compounds have shown anti-inflammatory activity; among them, phenolic compounds have attracted great attention due to both their large distribution among dietary components and the variety of biological activities that they display [9].

*Prunella vulgaris* var. *lilacina* is a perennial herb that is widely distributed in Korea, China, Japan, and Europe. *P. vulgaris* var. *lilacina* has been used as a traditional medicine to alleviate sore throat, reduce fever, and accelerate wound healing. In addition, this herb has been shown to have anti-inflammatory, anti-oxidant, anti-allergic, anti-microbial, anti-viral, and free radical scavenging activities [10,11]. *P. vulgaris* var. *lilacina* contains various compounds which are flavonoids, triterpenoids, phenolic acids such as campherol, rutin, rosmarinic acid, caffeic acid, and tannins that provide a great assortment of biological properties [12].

Several studies related to *P. vulgaris* var. *lilacina* have provided evidence that the ethanol extracts of this herb can suppress inflammation by inhibiting nitrite and prostaglandin E2 (PGE2) production by macrophages, suppressing the NF-κB activity [13–16]. Nevertheless, the studies have not yet considered the anti-inflammatory effects of the various solvent fractions of *P. vulgaris* var. *lilacina.* Thus, in this study, we focused on evaluating the effects of the various solvent fractions from the ethanol extracts of *P. vulgaris* var. *lilacina* in preventing inflammation. We also investigated a potential mechanism for the anti-inflammatory effect of *P. vulgaris* var. *lilacina* in association with NF-κB inhibition.

## 2. Results and Discussion

### 2.1. Effect of *P. vulgaris* var. *lilacina* on Viability of RAW 264.7 Cells

**Figure 1:**
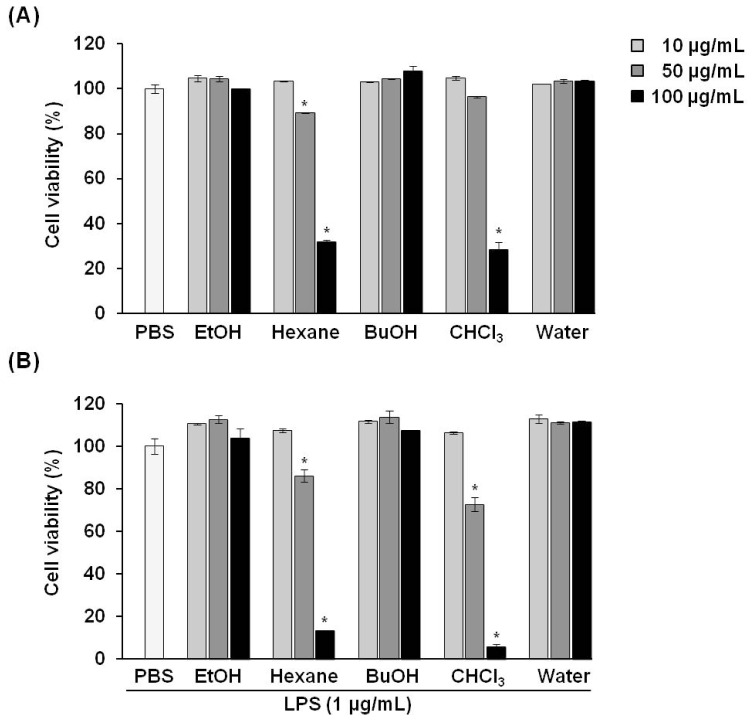
Effects of solvent fractions from *Prunella vulgaris* var. *lilacina* on the viability of RAW 264.7 cells. (**A**) Lipopolysaccharide (LPS) untreated; (**B**) LPS treated. Bars represent the mean and standard deviations from three different experiments performed in triplicate. *p < 0.05 significantly different from the LPS group.

The inhibitory effect of *P. vulgaris* var. *lilacina* on RAW 264.7 cell viability was determined by intracellular ATP content (Figure 1). Cells were treated with fractions of *P. vulgaris* var. *lilacina* at various concentrations (0, 10, 50, and 100 μg/mL) for 1 h and then co-incubated with lipopolysaccharides (LPS; 1 μg/mL) for an additional 24 h.

We estimated the influence on cell survival according to the following criteria: Cell viability values greater than 90% were considered unaffected by tested compounds, 80%–90% was modestly affected, and values less than 80% were considered affected by the cytotoxic effects of the compounds.

Our results showed that the various fractions of *P. vulgaris* var. *lilacina* had no cytotoxic effects on RAW 264.7 cells at concentrations of 10 μg/mL. For the hexane fractions (50 μg/mL), the viability of RAW 264.7 cells after exposure was 85%, which, according to our criteria, was a modest effect and was not considered cytotoxic in other reported studies [17–19]. In contrast, the group treated with chloroform (CHCl_3_) fractions resulted in a cell viability value of 73%. Therefore, except for the chloroform fractions, all fractions of *P. vulgaris* var. *lilacina* from 10 to 50 μg/mL were selected for subsequent experiments.

### 2.2. Effect of *P. vulgaris* var. *lilacina* on LPS-Induced NO and PGE2 Production

**Figure 2:**
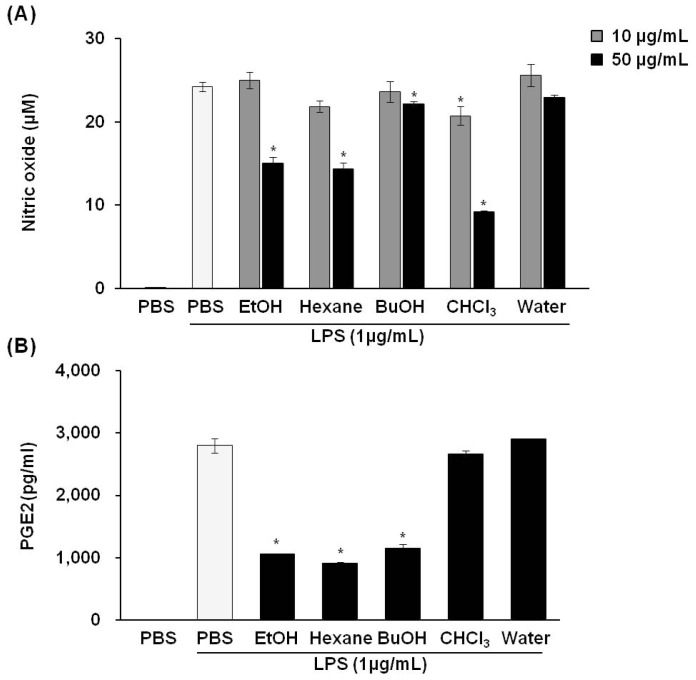
Effects of the solvent fractions from *Prunella vulgaris* var. *lilacina* on LPS-induced nitric oxide (NO) (**A**) and prostaglandin E2 (PGE2); (**B**) production in RAW 264.7 cells. Values show the means and standard deviations of three different experiments performed in triplicate. *p < 0.05 significantly different from the LPS-treated PBS group.

We next investigated whether *P. vulgaris* var. *lilacina* might have anti-inflammatory properties in LPS-stimulated RAW 264.7 cells. Various concentrations (0, 10, and 50 μg/mL) of *P. vulgaris* var. *lilacina* fractions were used on RAW 264.7 cells to test whether *P. vulgaris* var. *lilacina* could reverse LPS-induced accumulation of NO and PGE2. The results revealed that LPS (1 μg/mL) treatment for 24 h markedly increased NO and PGE2 production as compared with control group; however, *P. vulgaris* var. *lilacina* fractions significantly inhibited the production of these factors in a dose-dependent manner (Figure 2). In particular, NO and PGE2 secretion decreased by 40% and 60%, respectively, in cells exposed to the hexane fraction of *P. vulgaris* var. *lilacina*. For the chloroform fraction, NO secretion was strongly inhibited, suggesting that the chloroform fraction may influence cell death. Huang *et al*. [16] demonstrated that the ethanol extract of *P. vulgaris* inhibited LPS-induced PGE2 and NO production by 10%–35%. Our results are consistent with their reports. The remarkable aspect of our study is that we observed a stronger inhibition than their results. These results could be affected by various extracted compounds come from difference of composition of the solvent which were likely due to our using 70% ethanol for extraction instead of 95% ethanol as was used by the other group.

### 2.3. Effect of *P. vulgaris* var. *lilacina* on LPS-Induced iNOS and COX-2 Expression

**Figure 3:**
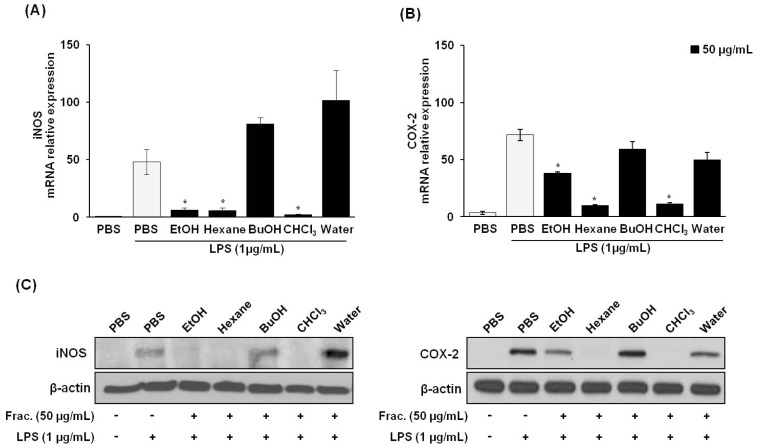
Effects of solvent fractions from *Prunella vulgaris* var. *lilacina* on LPS-induced pro-inflammatory mRNA expression and protein levels in RAW 264.7 cells. Cells were treated with fractions (50 μg/mL) and stimulated with LPS (1 μg/mL) for 12 h. (**A**,**B**) After incubation, cells were harvested for real-time RT-PCR to determine mRNA expression for (**A**) iNOS and (**B**) COX-2. (**C**) After incubation, cell lysates were used to determine iNOS and COX-2 protein levels via Western blot. Values show the means and standard deviations of three different experiments performed in triplicate. *p < 0.05 significantly different from the LPS-treated PBS group.

NO is acknowledged as a mediator and regulator of the inflammatory response and is made in large amounts by iNOS in activated inflammatory cells [20]. The enzymatic activity of COX-2 also can be influenced directly by NO and iNOS [21]. To elucidate the mechanisms of inhibition for the hexane fraction against NO and PGE2 production in LPS-stimulated RAW 264.7 cells, we assessed whether the inhibitory effects of *P. vulgaris* var. *lilacina* on these inflammatory mediators were related to iNOS and COX-2 protein and mRNA levels using western blot and real-time reverse transcription polymerase chain reaction (RT-PCR) analyses, respectively. The level of iNOS and COX-2 mRNA and protein expression was significantly elevated in macrophages treated with LPS. Treatment with *P. vulgaris* var. *lilacina* fractions attenuated LPS-induced iNOS and COX-2 expression (Figure 3). These results suggest that the inhibitory effect of the fractions on LPS-induced NO and PGE2 production was mediated by the inhibition of iNOS and COX-2 expression.

### 2.4. Effect of *P. vulgaris* var. *lilacina* Fractions on TNFα Production

**Figure 4:**
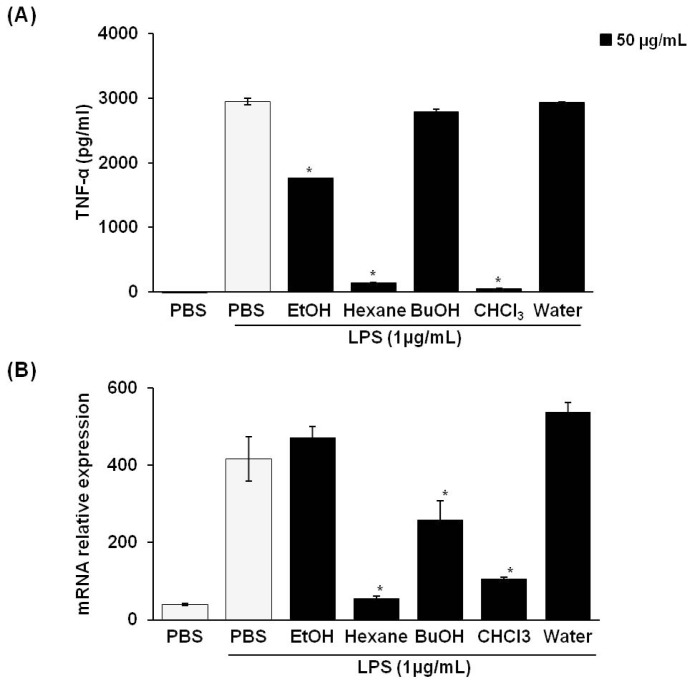
Effects of solvent fractions from *Prunella vulgaris* var. *lilacina* on LPS-induced TNFα levels (**A**) and TNFα mRNA expression (**B**) in RAW 264.7 cells. Cells were treated with fractions (50 μg/mL) and stimulated with LPS (1 μg/mL) for 12 h. Values show the means and standard deviations of three different experiments performed in triplicate. *p < 0.05 significantly different from the LPS-treated PBS group.

When macrophages are treated with LPS, the pro-inflammatory cytokine TNF-α is also increased and has an important role in the activation of NO and PGE2 [22]. Therefore, we examined the effect of treatment with *P. vulgaris* var. *lilacina* fractions on the mRNA expression and secretion of TNFα in LPS-stimulated RAW 264.7 cells. TNFα production was assessed in the culture supernatant by an ELISA. We found that TNFα was expressed at low levels in untreated controls (Figure 4). However, LPS stimulation significantly increased TNFα secretion at 24 h. The treatment with *P. vulgaris* var. *lilacina* fractions significantly suppressed LPS-induced TNFα secretion (Figure 4A). To evaluate whether the reduction in LPS-induced TNFα levels by *P. vulgaris* var. *lilacina* was due to the regulation of the TNFα gene in RAW 264.7 cells, we performed RT-PCR analysis. Similar to the results for TNFα secretion, *P. vulgaris* var. *lilacina* reduced the LPS-induced expression of TNFα mRNA (Figure 4B). These data indicate that *P. vulgaris* var. *lilacina* suppresses TNFα release at the transcriptional level.

### 2.5. Effects of *P. vulgaris* var. *lilacina* on NF-κB Activity and Translocation of NF-κB Subunits

The expression of iNOS and COX-2 requires the activation of NF-κB, which is an important mechanism for the overproduction of the inflammatory mediators in macrophages in response to LPS and cytokines [23–26]. NF-κB is located in the cytosol and is bound to the inhibitory IκB protein under unstimulated conditions. The activation of NF-κB in response to LPS stimulation leads to an increase in nuclear translocation and DNA binding ability.

**Figure 5:**
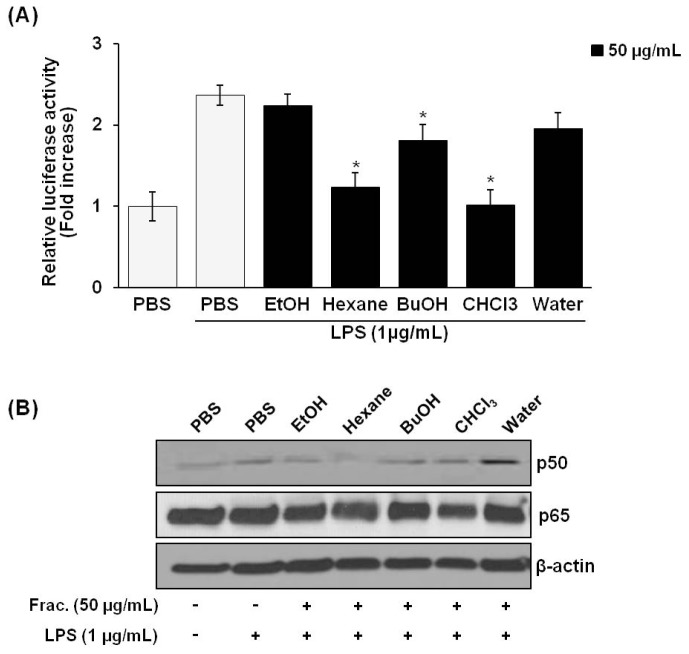
Effect of *Prunella vulgaris* var. *lilacina* on NF-κB activation as measured by the luciferase assay (**A**), and on nuclear translocation of the p65 and p50 subunits (**B**) in LPS-stimulated RAW 264.7 cells. Cells were treated with fractions (50 μg/mL) and stimulated with LPS (1 μg/mL) for 12 h. *p < 0.05 significantly different from the LPS-stimulated PBS group.

As shown in Figure 5A, treatment of *P. vulgaris* var. *lilacina* fractions (50 μg/mL) resulted in a significant decrease in NF-κB activation in LPS-stimulated RAW 264.7 cells as measured by using a firefly luciferase activity assay. Figure 5B shows the effect of *P. vulgaris* var. *lilacina* fractions on nuclear translocation of the p50 and p65 NF-κB subunits. The nuclear translocation of the p50 subunit was significantly reduced while the translocation of the p65 NF-κB subunit was only slightly reduced by the hexane fraction of *P. vulgaris* var. *lilacina*. No other fractions affected the nuclear translocation of p50 or p65 NF-κB.

Many studies have demonstrated that *P. vulgaris* var. *lilacina* extracts regulate NF-κB activation. According to Jun *et al*. [13] reported that the ethanol extract of the flower of *P. vulgaris* var. *lilacina* inhibited NF-κB activity by 50%, and Hwang *et al*. [14] reported that the water extract of *P. vulgaris* suppressed activation of the p65 NF-κB subunit. However, our results appear to differ from their results; we demonstrated that neither the ethanol nor the water extract of *P. vulgaris* var. *lilacina* affected the LPS-stimulated NF-κB activity. Instead, the hexane fraction of *P. vulgaris* var. *lilacina* inhibited both the p50 nuclear translocation and the NF-κB activity in LPS-stimulated cells. These results clearly demonstrate that unlike the ethanol extract of *P. vulgaris* var. *lilacina*, the hexane fraction has other anti-inflammatory components.

### 2.6. Identification of Components

**Figure 6:**
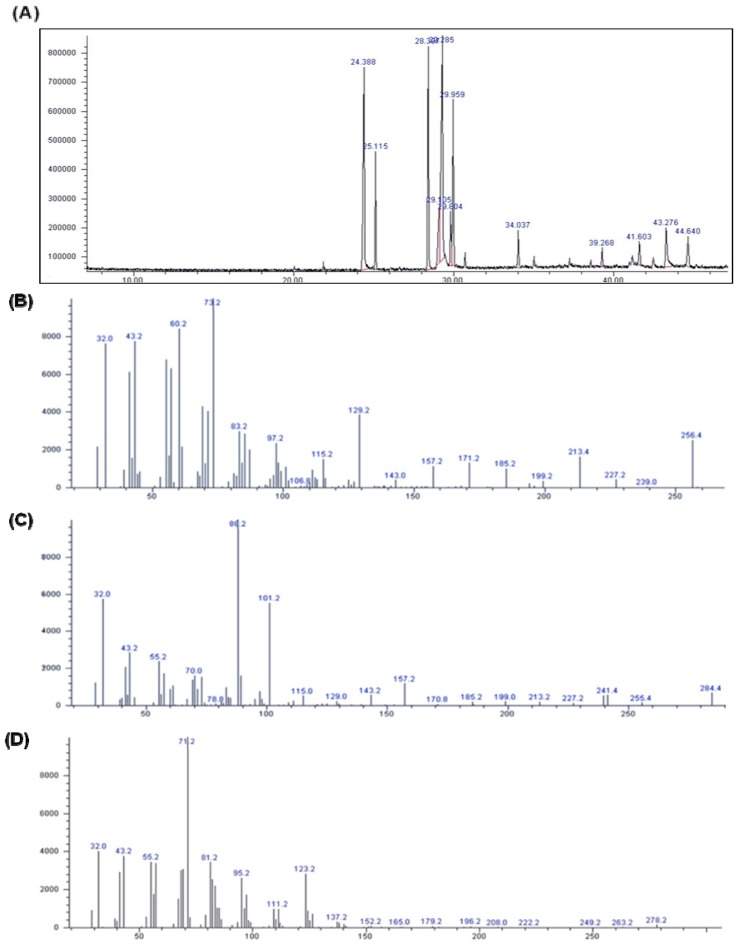

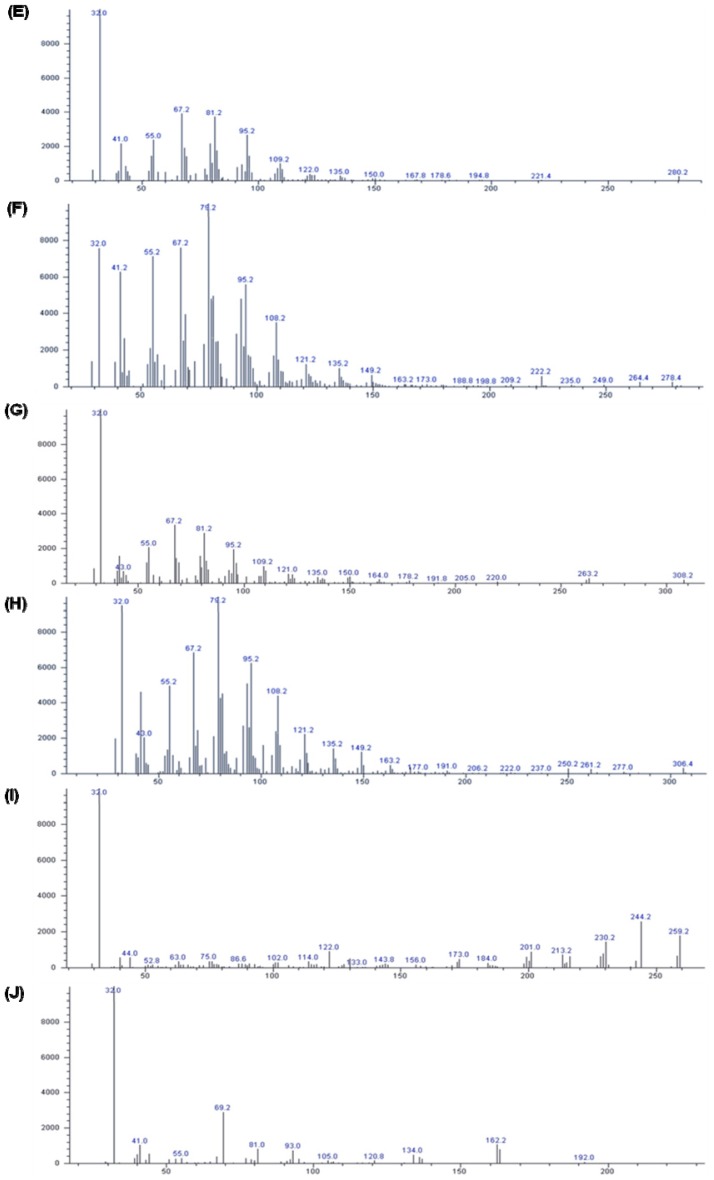

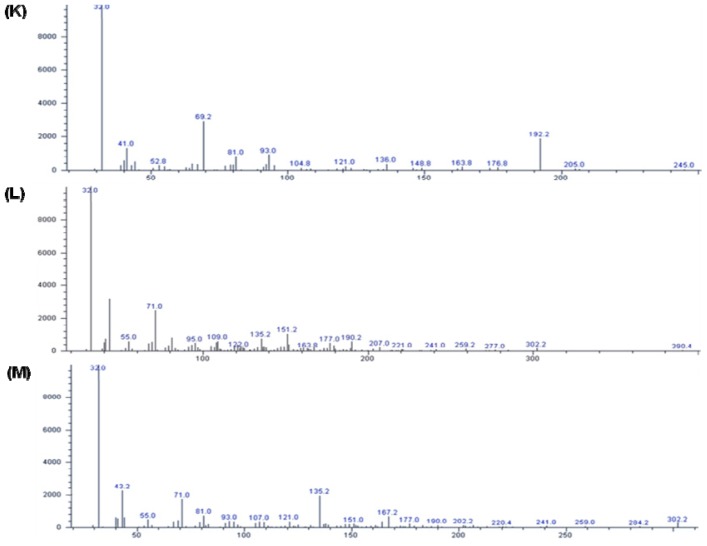
Gas chromatogram and MS Spectra of hexane fractions from *Prunella vulgaris* var. *lilacina*. (**A**) Gas chromatogram of hexane fractions from *Prunella vulgaris* var. *lilacina*; (**B**) hexadecanoic acid; (**C**) ethyl palmitate; (**D**) phytol; (**E**) ethyl linileate; (**F**) (*Z*,*Z*,*Z*)-9,12,15-octadecatrien-1-ol; (**G**) linoleic acid ethyl ester; (**H**) (*Z*,*Z*,*Z*)-ethy lester-9,12,15-octadecatrienoic acid, (**I**) 3-oxo-8,beta, *H*-eudesma-1,4,7(11)-trien-8,12-olide; (**J**) 3,7,11-trimethyl-2,6,10-dodecatrien-1-ol; (**K**) nerol; (**L**) linalyl formate, (**M**) 3-ethylenetricyclo[3.3.1.1(3,7)]decane.

We isolated various compounds from the hexane fraction of *P. vulgaris* var. *lilacina*: the identified compounds are shown in Figure 6. The spectrum profile from the gas chromatography-mass spectrometry (GC-MS) analysis confirmed the presence of 12 major components: hexadecanoic acid, ethyl palmitate, phytol, ethyl linileate, (*Z*,*Z*,*Z*)-9,12,15-octadecatrien-1-ol, linoleic acid ethyl ester, (*Z*,*Z*,*Z*)-ethyl ester-9,12,15-octadecatrienoic acid, 3-oxo-8,beta,*H*-eudesma-1,4,7(11)-trien-8,12-olide, 3,7,11-trimethyl-2,6,10-dodecatrien-1-ol, nerol, linalyl formate, and 3-ethylenetricyclo[3.3.1.1(3,7)]decane.

Several lipophilic compounds, including polyacetylenic acids, have been previously identified in the hexane fraction of *P. vulgaris* var. *lilacina* [27], and our research confirmed that this fraction indeed contained lipophilic compounds such as hexadecanoic acid, ethyl palmitate, and (*Z*,*Z*,*Z*)-ethyl ester-9,12,15-octadecatrienoic acid. It is well known that polyacetylenic acids have a therapeutic potential as antifungals and anti-oomycetes [27] while hexadecanoic acid, as an anti-inflammatory agent, has shown significant inhibitory activity against phospholipase A2 [28]. These reports are consistent with our results suggesting either that very potent unidentified anti-inflammatory compounds may be present in the hexane fraction of *P. vulgaris* var. *lilacina* or that hexadecanoic acid and other compounds may exert a synergistic effect on the inhibition of inflammatory mediator production in LPS-stimulated RAW 264.7 cells.

## 3. Experimental Section

### 3.1. Reagents

Dulbecco’s Modified Eagle’s Medium (DMEM), fetal bovine serum (FBS), and penicillin-streptomycin were obtained from Invitrogen (Carlsbad, CA, USA). LPS from Escherichia coli O55:B5 was purchased from Sigma-Aldrich (St. Louis, MO, USA). Antibodies against iNOS, COX-2, and β-actin were purchased from Cell Signaling Technology (Danvers, MA, USA). Monoclonal antibodies to p50 and p65 were purchased from Santa Cruz Biotechnology (Santa Cruz, Dallas, TX, USA). NF-κB-luciferase vector was purchased from Promega (Madison, WI, USA). All other chemicals were purchased from Sigma unless otherwise specified.

### 3.2. Sample Preparation and Extraction

*P. vulgaris* var. *lilacina* was purchased from the Plant Extract Bank (Dae-jeon, Korea). The dried *P. vulgaris* var. *lilacina* was ground into fine powders in a blender and was extracted three times with 70% ethanol. Subsequently, 10 g of 70% ethanol extract powder were weighed and dissolved in 500 mL distilled water and extracted stepwise with 500 mL of the following solvents: hexane, chloroform, and butanol. Each fraction was filtered through number 6 filter paper (Advantec MFS, Inc., Tokyo, Japan). The filtrates were combined and evaporated under vacuum and then lyophilized with a Bondiro Lyophpride freeze dryer (Ilshine Lab Co. Ltd., Dongducheon, Korea) at −70 °C under reduced pressure (<20 Pa).

### 3.3. Cell Culture and Viability

The RAW 264.7 macrophage cell line was purchased from the Korean Cell Line Bank (Seoul, Korea). RAW 264.7 cells were cultured in DMEM containing, 10% FBS and 1% penicillin/streptomycin at 37 °C in 5% CO_2_. To investigate cell viability of *P. vulgaris* var. *lilacina*, cells (1 × 10^4^ cells/well) were added to duplicate 48-well plates and incubated for 24 h, then treated with various concentrations (10, 50, and 100 μg/mL) of fractions of *P. vulgaris* var. *lilacina* for 24 h. Cell viability was measured with CellTiter Glo (Promega, Madison, WI, USA). The luminescent signal produced, which was proportional to the amount of ATP present in viable cells, was read on a Sirus luminometer (BertholdDetection System, Pforzheim, Germany). Cell viability is presented as the percentage of live cells in each well.

### 3.4. Nitrite Measurement

Nitrite was measured as an indicator of NO production after 48 h of treatment and LPS induction. The culture supernatant (100 μL) was placed in a 96-well plate, and an equal amount of Griess reagent (2% sulphanilamide and 0.2% *N*-1-(naphthyl) ethylenediamine dihydrochloride in 5% H_3_PO_4_) was added. The plate was then incubated for 10 min and the absorbance measured at 540 nm. The amount of NO was calculated using a sodium nitrite standard curve.

### 3.5. Prostaglandin E2 Measurement

After 12 h of treatment and LPS stimulation, the culture supernatant was collected. PGE2 was measured using a PGE2 ELISA kit following the manufacturer’s instructions (Abcam, Cambridge, MA, USA). Briefly, the diluted cell supernatant (100 μL) was placed in a 96-well goat anti-mouse IgG-coated plate and incubated for 2 h. After incubation, the plate was washed using the provided washing buffer, and the color was developed by adding PNPP (200 μL) substrate after 45 min. The amount of PGE2 was calculated by using a PGE2 standard curve.

### 3.6. Real-Time Reverse Transcription Polymerase Chain Reaction Analysis

To determine the expression levels of iNOS, COX2, and TNFα, real-time RT-PCR was performed using a real-time thermal cycler Qiagen rotorgene Q (Qiagen, Valencia, CA, USA) according to the manufacturer’s instructions. The cells were treated with *P. vulgaris* var. *lilacina* fractions and cultured for 12 h. Thereafter, cDNA was synthesized from the total RNA isolated from cells. The real-time PCR reaction was performed using 2× SYBR Green mix (Qiagen). All results were normalized to glyceraldehyde 3-phosphate dehydrogenase (GAPDH) expression. The following primer sequences were used for the real-time RT-PCR: GAPDH, 5′-GAG CCA AAA GGG TCA TCA TC-3′ (forward), 5′-TAA GCA GTT GGT GGT GCA GG-3′ (reverse); iNOS, 5′-AAT GGC AAC ATC AGG TCG GCC ATC ACT-3′ (forward), 5′-GCT GTG TGT CAC AGA AGT CTC GAA CTC-3′ (reverse); COX-2, 5′-GGA GAG ACT ATC AAG ATA GT-3′ (forward), 5′-ATG GTC AGT AGA CTT TTA CA-3′ (reverse); TNFα, 5′-AGC ACA GAA AGC ATG ATC CG-3′ (forward), 5′-GTT TGC TAC GAC GTG GGC TA-3′ (reverse).

### 3.7. Immunoblotting

Cells were lysed in RIPA buffer (150 mM Sodium Chloride, 1% Triton X-100, 1% sodium deoxycholate, 0.1% SDS, 50 mM Tris-HCl, pH 7.5, and 2 mM EDTA) on ice for 30 min. After centrifugation at 4 °C for 20 min (12,000 × *g*), the supernatant was collected. Protein concentrations were determined by BCA assay (GenDEPOT, Barker, TX, USA). Equal amounts of cell extracts were separated by 12.5% SDS-PAGE and transferred to polyvinylidene difluoride (PVDF) membranes (Bio-Rad, Hercules, CA, USA). The membranes were blotted with antibody and detection was performed with an ECL system (Pierce, Rockford, IL, USA) according to the manufacturer’s instructions.

### 3.8. Cytokine Determinations

The TNFα levels in the culture medium were determined by a Duo Set mouse TNFα ELISA kit according to the manufacturer’s protocols (R & D Systems, Minneapolis, MN, USA). The TNFα produced by LPS-treated cells was taken as 100%.

### 3.9. Transfection of RAW 264.7 Cells with pNF-κB Luciferase Vector

RAW 264.7 cells were transiently transfected with a pNF-κB-luciferase vector (Promega) using Lipofectamine 2000 (Invitrogen, Carlsbad, CA, USA) following the manufacturer’s protocol. Briefly, 5 × 10^4^ cells were placed in a 24-well plate and allowed to grow to 80%–90% confluency for 24 h. The cells were then treated with DNA-Transfast reagent mixture (50 μL) and incubated for 16 h. The amount of DNA added was 0.25 μg/well. After 16 h of incubation, each well was overlaid with 1 mL complete growth medium and the transfection was carried out for 48 h.

### 3.10. Measurement of NF-κB Luciferase Activity

After transfection, cells were treated with fractions (50 μg/mL) and LPS (1 μg/mL) for 24 h. Luciferase activity in the cells was measured using Dual-Luciferase Reporter Assay System (Promega) following the manufacturer’s protocol. Briefly, growth medium was removed and the cells were washed with 1 mL ice-cold PBS. After complete removal of PBS, passive lysis buffer (100 μL) was added and the plate incubated at room temperature for 15 min with shaking. After incubation, the luciferase activity was measured by adding cell lysate (20 μL) to the luciferase assay reagent (100 μL). Relative luciferase activity was determined by measuring the firefly luciferase activity and normalizing it to the Renilla luciferase activity.

### 3.11. Gas Chromatography-Mass Spectrometry Analysis

GC-MS analysis was carried out using an Agilent 6890 gas chromatograph equipped with a DB-5ms capillary column (60 m × 0.25 mm; coating thickness 1.4 μm) and an Agilent 5975 MSD detector (Loveland, CO, USA). Analytical conditions were as follows: injector and transfer line temperatures of 250 °C; oven temperature was programmed from 50 °C to 150 °C at 10 °C/min, from 150 °C to 200 °C at 7 °C/min, and from 200 °C to 250 °C at 5 °C/min; carrier gas helium at 1 mL/min; and split ratio 1:10. Identification of the constituents was based on comparison of the retention times with those of authentic samples. MS spectra of separated compounds were compared with one from Wiley 7 Nist 05 mass spectral database.

### 3.12. Statistical Analysis

Statistical analyses were performed with SPSS statistical software (version 12.0). The data represent means ± SEM from 3 independent experiments except where indicated. Statistical analyses were performed by using the Student’s *t*-test with *p* < 0.05 considered significant.

## 4. Conclusions

In conclusion, our observations support the hypothesis that the hexane fraction of *P. vulgaris* var. *lilacina* exerts anti-inflammatory effects by inhibiting the expression of LPS-stimulated iNOS and COX-2 inflammation-associated genes via suppression of transcription factor NF-κB activation. However, a limitation of our study is that our results were obtained by using cultured LPS-stimulated RAW 264.7 cells and, therefore, may differ from those results obtained *in vivo*. Thus, further studies will be required to investigate the anti-inflammatory effects of the hexane fraction of *P. vulgaris* var. *lilacina* in animal models of inflammation. In addition, the isolation and analysis of each anti-inflammatory compound from the hexane fraction of *P. vulgaris* var. *lilacina* is critical. Nevertheless, taken together, the results from our study suggest that the hexane fraction of *P. vulgaris* var. *lilacina* may be a potent anti-inflammatory therapeutic candidate.
